# The role of oral sodium bicarbonate supplementation in maintaining acid-base balance and its influence on the cardiovascular system in chronic hemodialysis patients – results of a prospective study

**Published:** 2016

**Authors:** C Voiculeț, O Zară, C Bogeanu, I Văcăroiu, G Aron

**Affiliations:** * Department of Internal Medicine, “Sf. Ioan” Clinical Emergency Hospital Bucharest, Romania; ** Clinical Department No. 1, “Carol Davila” University of Medicine and Pharmacy Bucharest, Romania; *** Department of Angiography, Catheterization, and Electrophysiology, “Sf. Ioan” Clinical Emergency Hospital Bucharest, Romania; **** Department of Nephrology and Dialysis, “Sf. Ioan” Clinical Emergency Hospital Bucharest, Romania; ***** Clinical Department No. 3, “Carol Davila” University of Medicine and Pharmacy Bucharest, Romania

**Keywords:** sodium bicarbonate, arterial stiffness, bone mineral disease, hemodialysis

## Abstract

**Background:** Major acid-base variations during dialysis and the imbalances in serum calcium levels intensified by them play a role in cardiovascular damage of hemodialysis patients. Early vascular walls modifications can be objectified by determining the pulse wave velocity (PWV) – a marker of vascular stiffness that is associated with increased risk of cardiovascular events.

**Material and methods:** This was a prospective study conducted on 63 chronic hemodialysis patients with diuresis above 500 mL/ 24 hours and predialysis blood pressure below 160 mmHg (treatment controlled) randomized in two groups for 12 months – the study group receiving interdialitic oral sodium bicarbonate doses and control group, without oral sodium bicarbonate supplementation, but receiving higher bicarbonate prescriptions in dialysis. All the patients were monthly evaluated by biochemical tests (serum calcium, phosphate, iPTH, bicarbonate), the assessment of prescribed doses of phosphate binders being undergone. Two PWV determinations and chest X-ray exams for coronary calcifications were done – at the beginning and end of the study for every patient.

**Results:** In the study group (n = 29), the mean age was 56.48 ± 12.78 years and the average duration of dialysis was 55.51 ± 34.53 months, the mean dialysis bicarbonate was 29.81 ± 1.41 mEq/ L and 27 of them (subgroup 0) had alkaline reserve (AR) 20-22 mEq/ L. The control group (n = 34) had a mean age of 57.35 ± 15.32 years and the mean dialysis duration 59.67 ± 34.79 months, with an average level of dialysis bicarbonate of 33 ± 2.2 mEq/ L necessary to maintain AR within guidelines. Depending on the mean AR obtained, this group was divided into three subgroups (subgroup 1, subgroup 2, and subgroup 3). There were statistically significant differences regarding the necessary of dialysis bicarbonate (p < 0.001), average serum calcium levels (p < 0.001) and serum phosphorus (p < 0.001), as well as PWV mean values and the number of vascular calcifications (p = 0.036) between the study and the control group. The average dose of phosphate binders was significantly higher in the study group (p = 0.01). At the end of the study, the serum iPTH average levels were decreased in the study group (p < 0.001) and significantly increased in the control group (p < 0.001).

**Conclusions:** Avoiding large variations in serum bicarbonate levels is an important step in hemodialysis patients’ management because wide acidosis-alkalosis variation can increase cardiovascular risks in terms of altering the vessel walls elasticity and favoring their calcifications.

**Abbreviations**:

GFR = glomerular filtration rate,PWV = pulse wave velocity, iPTH = intact parathyroid hormone,AR = alkaline reserve, BP = blood pressure,mEq = milliequivalents,L = liter

## Introduction

Chronic kidney disease involves many clinical conditions that have the irreversible decline in glomerular filtration rate as a common feature and the decrease in blood pH is one of the inevitable typical complications of this disorder.

Metabolic acidosis influences the nutritional status with important repercussions on the musculoskeletal system. It also has harmful effects on the functioning and structure of the cardiovascular system thus amplifying morbidity and mortality in uremic patients. Although hemodialysis is the most important method of correction for the metabolic acidosis of end stage renal disease patients, the changes in serum bicarbonate levels produced by a dialysis session are sometimes too abrupt and tempestuous, attracting adverse consequences. Large concentrations of bicarbonate used in dialysis solutions in order to maintain the alkaline reserve (AR) in the limits indicated by the guidelines, may induce serious metabolic alkalosis at the end of a dialysis session started with overt acidosis, and, these blood pH variations have influences on serum calcium levels, thus enhancing the risk of vascular and soft tissue calcifications [**1**-**5**].

The objective of this study was to highlight the repercussions of dialysis-induced acidosis-alkalosis variations on bone mineral metabolism parameters, as well as on vascular stiffness and vascular calcification process in chronic hemodialysis patients.

## Material and methods

The study was conducted over a period of 12 months, April 2014-April 2015, on a group of 63 chronic hemodialysis subjects selected from the patients of two dialysis centers in Bucharest.

The inclusion criteria were chronic kidney disease (CKD) stage 5 undergoing chronic hemodialysis (dialysis duration over 3 months), residual diuresis above 500 mL/day and predialysis blood pressure below 160 mmHg in the last 3 months (including controlled hypertensive patients). Patients diagnosed with diabetes, severe valve disease, systemic vasculitis, active neoplasia, and bone pathology diagnosed before the onset of chronic kidney disease were excluded from the study. 70 patients fulfilled these criteria and were listed for the study, of whom, 7 patients were lost along the follow-up and excluded from the results. The study had the approval of the Local Ethical Committee of the hospital and every participating subject signed an informed consent. Patients were randomized into one of two groups: study group – patients receiving oral interdialytic sodium bicarbonate (5 grams of sodium bicarbonate in the non-dialysis days) and the control group – patients who did not receive oral sodium bicarbonate supplementation.

Monthly predialysis serum calcium, phosphate, and bicarbonate levels taken before a mid-week session were recorded for every patientaccording to the dialysis monitoring protocol and by using a Mindray analyzer. iPTH was assessed at the beginning and at the end of the study for every patient by using ECLIA (Electrochemiluminescence Immunoassay). The 12-month average dose of phosphate binder prescribed for each patient was also recorded; the phosphate binders used in the dialysis centers participating in the study were calcium-based, containing 489 mg calcium carbonate per tablet.

**Fig. 1:**
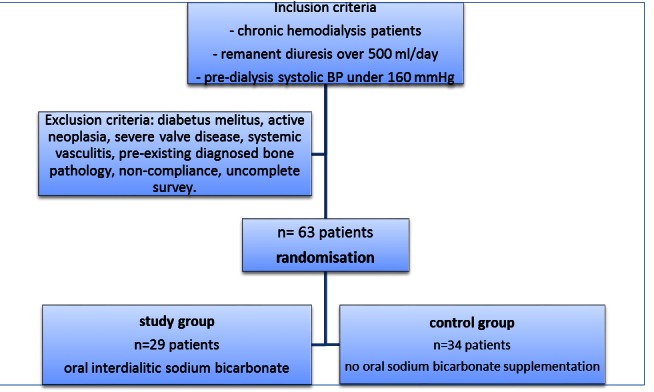
Study design scheme

Patients were additionally investigated, once at the beginning and once at the end of the study, by performing a chest X-ray in order to detectthe vascular calcifications and the PWV determination,so as to assess the elasticity of the vessel wall(after the measurement, the deviation from the normal maximum value of PWV was calculated for each patient, based on tables with normal aortic PWV included in the software on each age group and levels of BP). PWV evaluations were made by using Mobil-O-Graph (IndustrielleEntwicklungMedizintechnik, Germany) device and IEM-Hypertension Management embedded software. A scheme of the study design was drawn in **Fig. 1**.

Microsoft Excel was usedtocomplete the database; the data were statistically analyzed withSPSS by Mann-Whitney test, Pearson Chi-Square, Kruskal-Wallis test, Paired Samples T test, ANOVA test, Student T, Likelihood Ratio Test, and Fisher’s Exact. A p value < 0.05 was considered statistically significant.

## Results

63 patients were analyzed through the entire study period. There were 16 men among the patients who received oral intradialytic sodium bicarbonate in the study group (n = 29), the mean age beingof 56.48±12.78 years and the average duration of dialysis of 55.51±34.53 months. The 12-months average bicarbonate concentration in the dialysis solution was of 29.81±1.41 mEq/L in this group; 27 patients having the average AR in the guidelines limits of 20 to 22 mEq/L. The control group (n = 34 patients, 18 men) did not receive oral sodium bicarbonate supplementation, had a mean age of 57.35±15.32 years and a mean dialysis duration of 59.67±34.79 months; the average prescribed dialysis bicarbonate level being of 33± 2.2 mEq/L.

**Fig. 2:**
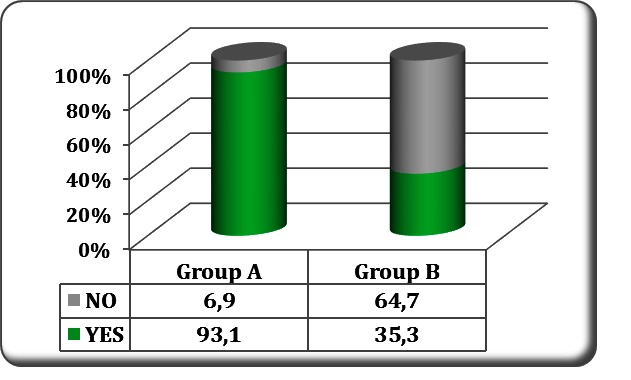
Patients with average alkaline reserve of 20 to 22 mEq/ L in the two groups

It was observed that the recommended AR levels for the hemodialysis patients, of 20 to 22 mEq/L, wasmaintained in most of the patients in the study group compared to only one third of the patients in the control group (93.1% versus 35.3%; p < 0.001, **Fig. 2**). The amount of bicarbonate in the dialysis solution was significantly lower in the study group (29.81±1.41 mEq/L versus 33±2.2 mEq/L; p < 0.001).

With respect to the AR average values obtained, the control group patients were divided in subgroups according to their 12-months average AR, and compared with the study group:

• subgroup 0 (study group) – patients who received oral sodium bicarbonate in interdialitic days – 29 patients

• subgroup 1 – patients in the control group with average predialysis AR 22 to 24 mEq/L – 8 patients

• subgroup 2 – patients in the control group with average predialysis AR 20 to 22 mEq/L – 12 patients

• subgroup 3 – patients in the control group with average predialysis AR below 20 mEq/L – 14 patients

**Fig. 3:**
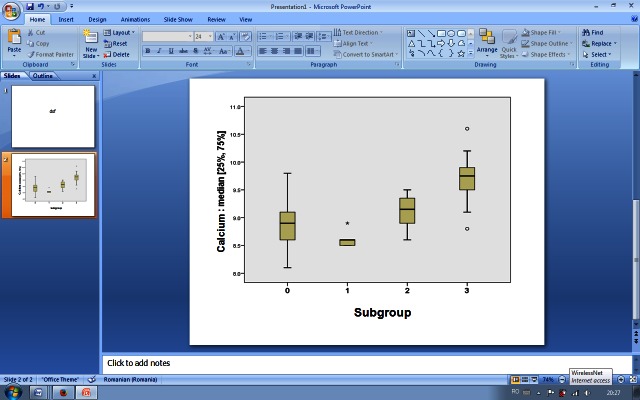
Median serum calcium levels distribution in the four subgroups

**Fig. 4:**
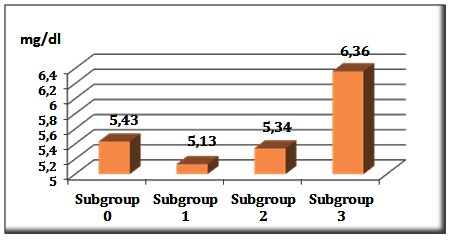
Mean serum phosphate levels distribution in the four subgroups

The X-ray results,the average values of laboratory tests and the average doses of phosphate binders recorded in each of the four subgroups are shown in Table 1, with statistically significant differences observed in terms of serum calcium, serum phosphate, doses of phosphate binders and number of vascular calcifications at the end of study (**Fig. 3**,**4**).

**Table 1:** Results recorded in the subgroups

Parameters	Subgroup 0 Study group	Subgroup 1	Subgroup 2	Subgroup 3	p value
		Calcium			
Mean ± SD	8.915 ± 0.414	8.600 ± 0.130	9.117 ± 0.228	9.707 ± 0.449	
Median [25%, 75%]	8.9 [8.6, 9.1]	8.6 [8.5, 8.6]	9.1 [8.9, 9.3]	9.7 [9.4, 9.9]	< 0.001
		Phosphate			
Mean ± SD	5.437 ± 0.604	5.138 ± 0.625	5.342 ± 0.995	6.364 ± 0.730	
Median [25%, 75%]	5.4 [5.3 5.8]	5.3 [4.9, 5.4]	5.5 [5.1 5.9]	6.5 [5.7 6.9]	< 0.001
		Phosphate binder			
Median [25%, 75%]	3.0 [0.0, 3.0]	7.0 [0.7, 9.7]	3.0 [0.7, 5.2]	6.0 [0.0, 10.0]	< 0.001
Vascular calcification (at the beginning)	44.4%	62.5%	41.7%	64.3%	0.509
Vascular calcification (at the end of study)	51.9%	75.0%	58.3%	92.9%	0.034

The control group required significantly higher average doses of phosphate binders than the study group: 4.5 [0.0, 9.0] versus 3.0 [0.0, 3.0] tablets/day; p = 0.016. However, it was observed that the subgroup 3, with pronounced acidosis, did not manage to compensate their serum phosphates upward trend and they had an average phosphate level upon the accepted limits (6.364±0.730 mg).

Variations in the serum iPTH levels in the study and the control group measured at the beginning and at the end of the study are shown in **Table 2**. There were differences between the initial and the final values in both groups but significant differences between the amount of iPTH variation in the groups, with a low decrease registered in the study group and a significant increase in the control groupwere also recorded (**Table 3**).

**Table 2:** Serum levels evolution of iPTH

Parameter	Study Group	Control Group
Initial iPTH Mean ± SD	583.402 ± 422.066	611.440 ± 475.143
Final iPTH Mean ± SD	550.281 ± 407.924	649.044 ± 469.771
p value	< 0.001	< 0.001

**Table 3:** iPTHdifferences between final and initial values in the study versus control group

Parameter	Group A	Group B	p value
		iPTH difference	
Mean±SD	-33.120± 26.982	37.604± 36.142	
Median [25%, 75%]	-28.5 [-41.8, -11.7]	37.0 [19.0, 58.7]	<0.001

No adverse events regarding the intradialytic sodium bicarbonate administration were reported. 7 patients were withdrawn from the study (6 in the study group and 1 in the control group) because of the non-compliance (2 cases) or the reduction of the diuresis below 500 ml/ day. The reduced number did not allow the performance of a statistical analysis between groups with respect to this mater. No hypertensive crisis was recorded in the study group.

The cardiovascular status was investigated by determining the PWV deviation and by performing chest X-ray twice in the study, at the same time with iPTH harvesting.

**Fig. 5:**
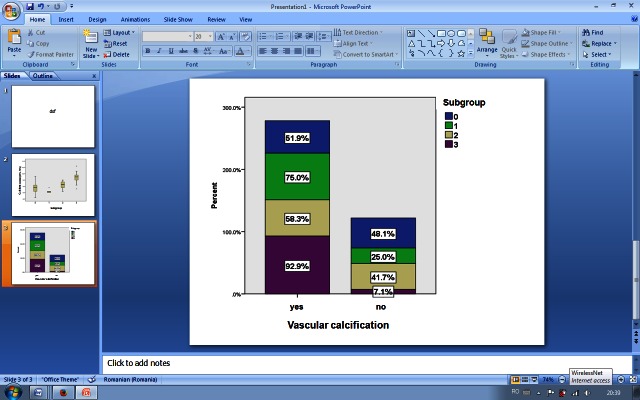
Vascular calcifications distribution at the end of study

Following PWV determinations, different trends of vascular elasticity were observed for each subgroup of patients, with the highest vascular stiffness seen in patients with the lowest AR average values (**Table 4**). Chest X-rays investigations showed new coronary arteries calcifications development in all subgroups. At the end of the study, the highest number of vascular calcification were objectified in patients with average predialysis AR below 20 mEq/L (**Fig. 5**) (at the beginning of study: 44.4% – subgroup 0; 62.5% – subgroup 1; 41.7% – subgroup 2; 64.3% – subgroup 3; p = 0.509; at the end of study: 51.9% – subgroup 0; 75.0% – subgroup 1; 58.3% – subgroup 2; 92.9% – subgroup 3; p = 0.034).

**Table 4:** PWV evolution

Subgroup	PWV initial Mean ± SD	PWV final Mean ± SD	p value
0 (n = 27)	8.826 ± 5.095	8.115 ± 4.329	0.070232
1 (n = 8)	9.225 ± 5.837	9.963 ± 6.496	0.196111
2 (n = 12)	8.450 ± 6.263	9.967 ± 6.216	0.000596
3 (n = 14)	17.336 ± 4.254	22.100 ± 5.553	0.000012

## Discussions

In this study involving chronic hemodialysis patients, two different methods of compensating the characteristic metabolic acidosis were applied: raising the bicarbonate levels in the dialysis solution (in the control group) versus temperate bicarbonate levels in dialysis and interdialitic oral sodium bicarbonate supplementation (in the study group). Consequently, there was a significant higher amount of bicarbonate used in the dialysis sessions of the control group compared with the study group (29.81±1.41 versus 33±2.2 mEq/L, p < 0.001), the AR was much better preserved in the study group (93.1% patients versus 35.3% patients within recommended limits) and, there were differences regarding the evolution of the vascular stiffness (PWV values) and the prevalence of coronary artery calcifications in comparison with the study group,even in the subgroups with a correct average AR.

A significant difference was also observed between the percentages of vascular calcification found in the four groups gathered according to AR average values obtained in the study period. The lowest prevalence of the coronary calcifications was noted in patients who received oral interdialytic bicarbonate and maintained their predialysis AR between 20 to 22 mEq/L (subgroup 0). Moreover, a small but significant increase of 6.4% was observed in patients with the same AR (20-22 mEq/L), but kept in limits by using a greater amount of bicarbonate in dialysis (subgroup 2), followed by patients with predialysis average AR 22 to 24 mEq/L (subgroup 1) and culminating with the calcifications objectified in subgroup 3, AR below 20 mEq/L (51.5%, 58.3% 75% and 92.9%; p = 0.03).

Acidosis causes buffer systems activation involving the accumulation of serum calcium and phosphate ions through bone resorption, with major influence on vascular endothelium and an important role in the development of vascular calcifications [**3**]. In addition to that, acidosis-alkalosis variations are extremely harmful for the whole body, which continually tries to adjust blood pH with questionable adaptive methods.

The harmful effects of acidosis on the cardiovascular system were also highlighted on 107 patients in dialysis, in the study by Oka et al. in which they observed a negative correlation between serum bicarbonate levels and coronary artery calcification [**6**]. Other studies described the lowest mortality risk in dialysis patients with predialysis AR values maintained inside the 20-22 mEq/l limits [**2**,**7**]. Patients in our study also met the best biochemical and paraclinical results in this serum bicarbonate values interval. What should also be noted is that there were two subgroups of patients with these values of AR (the study group and subgroup 2), the differences between them being made by the amount of bicarbonate used in the dialysis sessions and the variation of blood pH that it produced.

The administration of oral intradialytic bicarbonate has the advantage of a constant buffering acidosis to avoid fluctuations of pre-dialysis and postdialysis AR. The increase in the dialysis solution bicarbonate levels buffered acidosis is prone to alkalosis after the dialysis session, which is proved to be thecontributing factor to hemodynamic instability and QT interval elongation [**8**-**12**].

Patients who underwent hemodialysis with higher amounts of bicarbonate prescribed for keeping the predialysis AR in the recommended limits, experienced acidosis-alkalosis games with important repercussions on the cardiovascular system, because these games involved variations in calcium and phosphate serum levels in order to obtain a buffer acidosis. The highest levels in serum calcium and phosphate were recorded in patients with predialysis AR below 20 mEq/L (9.70±0.44 mg/dL; p < 0.001 for serum calcium, 6.36±0.73 mg/dL; p < 0.001 for phosphate).

The results of this study are in agreement with the literature database, highlighting the negative impact of low predialysis serum bicarbonate levels (predialysis AR below 20 mEq/L) but also of wide acidosis-alkalosis variations on mineral metabolism and cardiovascular system [**13**,**14**].

Because the body uses calcium to balance pH, patients may develop false hypocalcaemia. Therefore, true level of serum calcium cannot be assessed in those with variations in pH and there is the risk of extra calcium load, thereby promoting vascular calcification. The highest doses of calcium phosphate binders were administered to patients with serum bicarbonate below 20 mEq/L and to thosewith predialysis AR 22 to 24 mEq/L (6 [0.10] and 7 [0.7, 9.7] tablets/day; p < 0.001).The serum calcium level should be determined by bringing the AR to normal levels or adjusted according to it.

Although the prescription for phosphate binders was high in subgroups 1, 2 and 3, the highest serum phosphates levels were registered in patients gathered in the subgroup with an average AR below 20 mEq/l. Phosphate is considered a vascular toxin, having an important role in the uremic and even in the non-uremic vascular disease [**15**-**18**].

The fact that the cardiovascular disease represents a common pathology in chronic hemodialysis patients and that there is a strong influence of mineral bone disorders and acid-base imbalance in the magnitude of the vascular repercussions, were emphasized once again in this study. Therefore, correcting such imbalances allows the prevention of major cardiovascular events [**19**,**20**].

## Conclusion

Alkaline reserve is one of the most important parameters in the management of chronic hemodialysis patients, which can be simply and efficiently corrected by the intradialytic oral administration of sodium bicarbonate, avoiding large variations in blood pH and thus in calcium, phosphate, and iPTH levels that favor the alterations of the cardiovascular structures.

**Conflict of Interests**

The authors declare that there is no conflict of interest regarding the publication of this paper.
